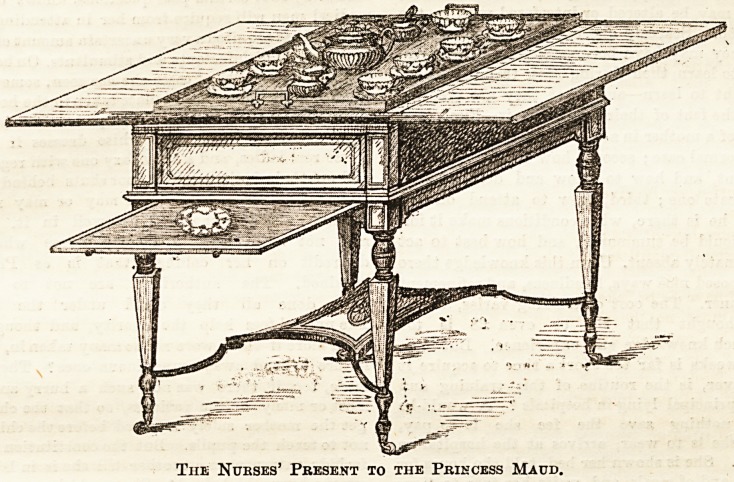# The Hospital Nursing Supplement

**Published:** 1896-02-29

**Authors:** 


### "The Hospital" Nursing Mirror.

Being the Extra Nursing Supplement of "The Hospital" Newspaper.

[Contributions for this Supplement should be addressed to the Editor, The Hospital, 428, Strand, London, W.C., and should have the word "Nursing" plainly written in left-hand top corner of the envelope.]

### News from the Nursing World.

### NEW HOME FOR NURSES AT ST. THOMAS'S HOSPITAL.

The opening of two of the hitherto closed wards at St. Thomas's has, of course, necessitated an increase in the nursing staff, and the charming quarters which have been prepared in what used to be the apothecary's house by way of additional accommodation met with universal admiration at the opening on Friday, February 21st. Eighteen nurses and sisters can be housed there, and the rooms are everything that the most ardent advocate of comfort for nurses could desire. There are three bed-sitting-rooms for sisters, pleasant rooms furnished with every regard to good taste in the matter of carpets and chintzes, and the other little details which mean so much to the occupant. Each nurse has a separate bed-room, and there are excellent bath-rooms and lavatories. The house is provided throughout with electric light. Each bed-room is very completely furnished with a hanging cupboard, with mirror, a combination dressing-table and chest of drawers, and wash-hand stand. The floors are everywhere covered with kamptulicon, and there are small pieces of carpet in the bed-rooms. The sitting-rooms are carpeted. The arrangements are altogether quite an example of what can and ought to be done in this way for the comfort of nurses when extension of existing accommodation is proposed.

### MEDICAL WOMEN IN SCOTLAND.

In the first year of the existence of the Scottish Association for the Medical Education of Women the students entering for the winter session (1889-90) numbered fifteen, for the corresponding session of 1895-96 they reach the respectable figure of 64, and now the question of establishing a building in connection with the Association will have to be seriously considered. Recently new practical anatomy rooms have been added, to the great advantage of the students.

### NURSES' HOME, DUNDEE.

The new Nurses' Home in connection with the Dundee Royal Infirmary will apparently be a roomy and comfortable one, quite in the most approved sense of those terms. The intended site is between the infirmary buildings and the boundary wall on the west, just to the north of the main entrance. The home, which is to be erected from plans by Mr. Alexander Johnston, of Dundee, will be in the form of the letter L, with a frontage to the south of 94 ft. and to the west of 68ft.; it will be three-storeyed, containing in all some thirty-two bedrooms, with library and sitting rooms, and plenty of bathroom and lavatory accommodation. No open fires are provided in any of the rooms. The money for the erection of the home has been provided by Mr. W. Ogilvy Dalgleish, of Errol Park, who has increased his original gift of £3,500 to £5,000.

### AN EXAMPLE TO COTTAGE HOSPITALS.

A meeting of the committee of the Tilbury Cottage Hospital was held a week or two ago, at which the constitution of the future nursing staff came under consideration. It was decided to appoint a matron at a salary of £50 per annum, rising £5 a-year to £60, and two nurses at a salary of £25 each, the hospital to pay half their premiums (£6 respectively) to the Royal National Pension Fund for Nurses. We gladly welcome the evidence thus afforded that the wisdom of such a provision for the future is being more widely recognised every year amongst every class of charitable institution, and warmly congratulate the committee upon their action. The Tilbury Cottage Hospital shows an excellent example to all similar hospitals throughout the country.

### CHERTSEY BOARD OF GUARDIANS.

The Workhouse Infirmary Nursing Association have been most reluctantly compelled to withdraw their nurses from the Chertsey Workhouse Infirmary, and the reasons for this step will be made public in due course.

### THE SARAH ACLAND HOME.

A largely attended meeting on behalf of this home was recently held in the hall of Exeter College. Sir Henry Acland, who was present, cannot fail to have been gratified by the universal testimony borne to the valuable work which is being done in Oxford through the home, which perpetuates the memory of his wife. An excellent speech was made by the Dean of Christ Church, who presided, in which he spoke of the educational value of all good and thorough work, adding that he was "not afraid to say he had never come across any work so well done as was the work of a really perfect nurse." Mrs. Dacre Craven spoke at some length, and Mrs. Liddell, who has been for seventeen years the president of the home, asked for special help towards certain necessary repairs and alterations in the home; £100 had been collected, but some hundreds would be needed, and she thought that this money for their district nurses ought to come from Oxford itself.

### DISTRICT NURSES AT RYDE.

At the recent meeting of the Ryde District Nursing Society, it was shown to be in an eminently satisfactory condition. Much sympathy was expressed by the various speakers with both the Queen and Princess Beatrice, the latter of whom had been present at the last annual meeting with Prince Henry of Battenberg. The Princess became president of the society three years ago, and it was affiliated with the Queen's Jubilee Institute in the same year.

### DISTRICT NURSING IN BIRMINGHAM.

The Birmingham District Nursing Society celebrated its twenty-fifth birthday on February 13th, when the Mayor presided at the annual meeting. The society is affiliated with the Queen's Jubilee Institute, and the consequent annual inspection is much valued by the committee. The chairman called attention to the vast amount of work done by ten nurses for a yearly expenditure of less than £1,000, and suggested that the thought of how much more might be done with a larger staff and income should stir up the public to do its part by doubling or trebling the resources at command. The branch home at Saltley, although its success had not quite fulfilled expectations, yet had been proved to fill a much-felt want, and it has been decided to continue the work there for at least another year.

### DISTRICT NURSING IN HOLLAND.

Miss Kruysse, who was the pioneer of district nursing in Holland, and received her training in that branch of nursing at St. Patrick's Home, Dublin, an affiliation of the Q.V.J.I., starting work on similar lines at Zwolle in 1894, reports very encouraging progress. A little later the system was started at Amsterdam, and Miss Kruysse is now preparing to plant it in Rotterdam. The scheme is warmly supported by the Queen Regent.

### EXPLOSION AT JOHANNESBURG.

The recent terrible explosion at Johannesburg strained the resources of the hospital to their utmost limit—and beyond, so far as space capacity went—over two hundred injured persons being brought in in the course of the evening. The beds were filled by the first arrivals, extemporised beds on the floor having to be made up for those coming in later, and finally an overflow infirmary was organised at the Wanderers' Athletic Club's headquarters. There seems to have been plenty of medical aid at once at hand; doctors, nurses, and lay volunteers worked with a will, and a hundred of the slighter cases were able to be discharged as soon as their wounds were dressed. Four deaths occurred amongst the wounded in the hospital soon after their admission. The distress caused by this wholesale destruction in the poor part of the town will be very widespread, but subscriptions opened at once have already reached a very large sum, £60,000 being subscribed by ten o'clock on the very evening of the explosion.

### MIDWIFE AND NURSE.

"The mothers" of Bognor have been much perturbed by, and inclined to protest against, the recent action of the committee charged with the superintendence of the district nursing in that town, in replacing the present district midwife by a nurse who is to be both a midwife and a trained nurse. At the annual meeting the chairman explained that "a more general nurse" was greatly needed, and while it was natural that those to whom Nurse Northcote had endeared herself by much kindness should be sorry to lose her, the committee felt the change was quite necessary, as funds would not admit of two nurses being engaged. It is very encouraging to note how the need for thorough and general training in district work is being realised and provided in all parts of the country.

### PROBATIONERS WANTED.

Many women who nourish the wish to enter a London hospital for training are deterred from making applications at such and such an institution because they hear or see it stated that "out of 2,000 or more applications yearly only one or two hundred are accepted." But this announcement should not discourage really suitable candidates, for perhaps only those behind the scenes can form an adequate idea of the impossibility of three-fourths of those applicants. By the time the "2,000" would-be candidates are weeded out the actual number of suitable ones left may fall below that of the vacancies to be filled, and still there is the chance that the month's trial may result in failure for some of the selected. Therefore, applications from women who are really well qualified to enter the nursing profession are ever welcome to matrons anxious to maintain a high standard on their staff. At the London Hospital this is certainly the case, and Miss Lückes is glad to hear from any number of candidates fulfilling the necessary requirements as to age, health, and education. A great deal of quite unnecessary trouble is given every year to the busy heads of training schools by thoughtless people, who do not seem to understand plain English, and return the forms sent to them on application with replies which obviously put them out of court, which the exercise of a little common-sense would have obviated.

### SHORT ITEMS.

The quarterly meeting of the Society for Providing Nurses for the Sick Poor of Dublin was held on the 4th inst., at the Depôt, College Square, Mrs. McNeile, the president, in the chair.—More than fourteen thousand visits were paid last year by the four nurses of the Southport District Nursing Society.—A successful dance was held at the Town Hall Assembly Rooms, Cardiff, on February 17th, in aid of the Cardiff Branch of the Queen's Jubilee Institute for Nurses.—On account of her departure from St. Anne's, Mrs. McKerrow has, to the great regret of all concerned, resigned the office of secretary to the St. Anne's Ladies' Sick Aid and Nursing Society, on behalf of which she has laboured most energetically.—A good deal of excitement has lately prevailed amongst the staff at Kew Gardens owing to the importation from Swanley Horticultural College of two lady gardeners.—The Bishop Auckland District Nursing Association has had a year of successful work. There is now a small balance in hand, instead of a deficit, as was the case last year.—A well-attended concert in aid of the local District Nurse Fund was given in Truro last week.—The annual general meeting of the Society for the Protection of Birds was held at the Westminster Palace Hotel, Victoria Street, on the 20th inst. The Duchess of Portland is the president.—The Bill to render women eligible as Poor-law Guardians in Ireland came on for discussion in the House of Commons on Tuesday in last week, and was read a second time by a majority of 264, only eight members voting against it. The Bill was read a third time on the following day.

### Lectures on Nursing.

BY A SUPERINTENDENT OF NURSES.

### XI.—TYPHOID FEVER.

ANOTHER disease in the treatment of which feeding plays an important part is typhoid fever. It is generally contracted by drinking impure water derived from decaying organic matter, such as the infiltrations from cesspools or latrines, and containing in suspension the germs of the disease. The outbreak at Worthing some years ago, of which we heard so much, was the result of the drainings of a sewage farm dripping into a newly-sunk well which largely supplied the town with water, and the epidemic was of a particularly virulent form. We often hear of "milk epidemics," and when these occur there has usually been a case of typhoid at a farm house or dairy, and through some defective sanitary arrangements the water used at the farm has become infected by the excreta, and has been used either for adulterating the milk or for "washing the pans," and so has contaminated the milk and carried the disease to a large number of people. Some doctors insist on all milk being boiled before being given to their patients, so that the germs may be destroyed.

The incubation period of typhoid usually lasts from ten to fourteen days. The onset of the disease is very insidious. The rash appears from the seventh to the fourteenth day, and consists of slightly raised rose-coloured spots about the size of a pin's head. They appear in crops, disappear on pressure, to reappear when the pressure is removed. Each spot lasts about four days. In typhoid fever there is ulceration of the small flat raised patches, known as Peyer's patches, in the small intestine. During the first week these patches become inflamed. During the second week they slough, and in the third week the slough comes away, leaving an ulcer. This is the most dangerous time, owing to the wall of the intestine being so thin from the separation of the slough, and because the patient feels so much better that he begins to beg eagerly for solid food, and it is difficult to persuade him that he must on no account take anything but liquids.

The general symptoms of typhoid are tenderness over the abdomen, diarrhœa (though sometimes constipation), the motions being like pea soup in colour and consistency, and the reaction alkaline turning red litmus paper blue. In case of constipation it should be remembered, should an enema of soap and water have been administered, that in itself would be sufficient to render the stools alkaline. This possible source of fallacy should be borne in mind by the nurse when testing. Bleeding from the nose frequently occurs.

At the end of the first week the temperature will probably reach 103 deg., or even 105 deg. in the evening, and will most likely be 1 or 1½ deg. lower in the morning. In the third week the temperature gradually begins to go down if the case is progressing favourably. This is called "lysis," but if the temperature suddenly falls, it is looked upon as a very unfavourable symptom, as it denotes hæmorrhage or perforation, and the danger is very great.

If sordes collects on the teeth, lips, or tongue, the nurse should wrap a piece of lint round the finger dipped in Condy's fluid or carbolic lotion (1 in 40) and gently rub the teeth, and the mouth can be wiped out with tiny swabs of cotton wool dipped in some disinfecting fluid and held by dressing forceps, the pledgets always being burnt immediately afterwards.

The nurse should be very particular never to allow a typhoid patient to sit up, because the intestines being more or less ulcerated, the slightest pressure put upon them may cause perforation. At the same time, she should not permit him to lie flat on his back too long together for fear of pneumonia. If he is constantly in this position the lungs do not get full play and the blood is not aerated, so that the base of the lungs become congested and pneumonia sets in. The patient should, therefore, be moved gently right on to his side, care being taken to move him by his shoulders or thorax and by his hips, so as to avoid touching the abdomen, or, if by way of changing the posture, not right on to the side, pillows should be placed under his back so as to support it on one side. The temperature will have to be taken every four hours, perhaps oftener, and any sudden rise or fall should be at once reported. The patient may have to be sponged with cold, iced, tepid, or hot water. Sometimes packs are ordered, and in some hospitals the bath treatment is employed, and the patient is allowed to remain for days together in the bath, that the temperature may be kept down and the poison eliminated from the system as much as possible through the pores of the skin. In other cases Leiter's tubes are used. Before attempting to fill these the air should be exhausted by means of a syringe, and they should be kept under water till the circulation is complete. It is a good plan to make a wide flannel binder, with loose shoulder straps, to line the front of the binder with calico, leaving the lower edge open so that the tubes can be kept in position, even if the patient is somewhat restless. The binder can be turned back to front if the tubes are ordered for the back. The tubes should never be put on without calico, lint, or muslin being put next to the body. A cradle can be used to prevent the weight of the clothes resting on the patient, and in case of an ice-bag being ordered, the weight should be mainly borne by the bars of the cradle, from which it should be suspended, so as just to touch the surface of the part to which it is applied. In some hospitals ice is kept in small toy buckets which are hung inside the cradle.

In case of distension enemata are ordered, or the long rectal tube may have to be passed for four or five inches, and if that is insufficient, for double that length. Enemata serve a threefold purpose—to empty the rectum, for nutritive purposes, and to check diarrhœa.

The nurse should keep her patient scrupulously clean, both in person and in linen, and should guard against bed sores by rubbing the back night and morning with spirit; zinc ointment may be used if there is much moisture, or if sores have formed. Saturated solution of tannic acid in spirit is an excellent thing. All vessels used for feeding the patient, bed-pans, and other utensils should be kept apart from others with some distinguishing mark upon them; those used for receiving the excretions should always have some disinfecting solution inserted previous to use.

All linen coming in contact with the patients should be put at once into a covered pail and be saturated with disinfecting fluid; if carbolic is used, it should be one in twenty to be of service. Every typhoid stool should be disinfected before it is disposed of. As the chief danger of infection lies in the excreta, the nurse should wash her hands and dip them into a disinfectant after attending to the patient, taking great care that her nails are thoroughly cleansed.

The feeding of the patient is a very important matter, as the slightest indiscretion may produce fatal results. The following diet scale has been found of great use in one hospital:—

FLUID DIET DURING THE DIFFERENT STAGES OF THE FEVER.

| DIET. | REMARKS. |
|---|---|
| Milk with soda or barley water. | Three-parts of milk with one-part of soda water or strained barley water, three pints in 24 hours; generally given half a pint every 4 hours. |

| | |
|---|---|
| Lemon or orange juice, with water. | Strained, one pint in every 24 hours. |
| Iced water. | |
| Weak tea, cocoa, or coffee. | |
| Eggs. | Two in 24 hours, yolks only, lightly beaten, and added to milk or tea. |
| Beef tea and chicken broth. | Strained; one pint and a-half in 24 hours. |
| **Thickened Fluids.** | |
| Gruel. Arrowroot. | |
| Benger's food. | |
| Raw beef juice. | |
| Brand's essence. | |
| **Semi-solids.** | |
| Calf's-foot jelly. | Jelly may be frozen. |
| Milk shapes. Cream. | |
| Custard. Chocolates. | Chocolates are very grateful to a patient who is craving for solid food. |
| Poached or lightly boiled eggs. | |
| **Solids.** | |
| Thin bread and butter without crusts. | |
| Milk puddings. | |
| Fish (plaice). | |
| Mince. Chicken. | |
| Fancy diet. | |
| Full diet. | |

Soda water must never be given effervescing for fear of its causing distension.

During the convalescent stage food must be given frequently, but in small quantities, so as not to overload the stomach.

Great care will have to be taken to guard against relapse, and the patient must not be allowed to exert himself too much, and must be kept from exposure to cold.

Inflammation of the veins (phlebitis) sometimes comes on during the convalescent stage. It begins frequently with pain in the groin, followed by swelling of the legs. It is then necessary for the patient to maintain the recumbent position for some little time.

### Workhouse Infirmary Nursing.

A perusal of the annual report of the Workhouse Infirmary Nursing Association shows how trying the work in poor law infirmaries often is, and how very different are the surroundings to those to which nurses have usually been accustomed during their training.

In the large old-fashioned buildings the difficulty often is the overwork; not only from excess of patients, but from bad arrangements. The wards may be far from one another, the nurses may have to cross ill-paved yards in all weathers, and this not by day only but at night. The wards are often crowded, conveniences and necessaries are few, and sanitation bad. Moreover, the wards for the sick are too often mixed up with those for the able-bodied, which adds greatly to the difficulty of keeping order. Under such circumstances supervision is difficult, and the work is not only hard but disheartening.

In small lonely country infirmaries the difficulties are of a somewhat different nature. The nurse is lonely and her occupation is often dull. Of actual active nursing there is not much, and to fill up time a good deal of work outside her proper sphere often falls to her share, such as supervising the bed-making and bathing of the able-bodied, the personal cleanliness of the older inmates, and even of tramps and casuals, and the oversight of the nursery. Then there is often a terrible lack of the most elementary nursing necessaries, and a great trouble in the country is the lack of water, for in some country infirmaries neither hot nor cold water is laid on, the cold having to be carried in buckets from the main building, and hot water being quite an unusual luxury.

Then there are difficulties with the officials, to overcome which the greatest tact is often necessary, and in addition there is the fact that a certain amount of moral influence is expected from a nurse, which is somewhat foreign to her training. In these out-of-the-way places the patients are often dependent on the nurse for the only bit of cheeriness and comfort in their monotonous lives, and she has it in her power by sympathy and tenderness greatly to brighten the latter days of many aged and suffering people.

In many ways, then, a nurse taking up infirmary work may find herself in positions in regard to which her purely hospital training may give her but small clue as to the best course. Accustomed to perfect appliances, ample water supply, automatic sanitary arrangements, plentiful linen, and complete surgical antisepsis, work is a puzzle where these are absent, and too often is it the case that probationers are so held back from all sympathetic relations with the patients, such being reserved for the "sisters," that from mere want of training in its exercise any display of personal sympathy becomes difficult without risking a loss of discipline.

While, then, every nurse should undergo a part of her training in a well-appointed, active hospital, those who wish to undertake infirmary work would do well to attach themselves for part of their time to some workhouse infirmary, at which they might gain some knowledge of their new duties before undertaking the complete responsibility which will be thrown upon them.

### Pirated Advertisements.

We have more than once called attention to the republication by the *Nursing Record* of advertisements taken from our columns without acknowledgment. Two notable instances have just come before us.

On February 8th, 1896, we published an advertisement for a probationer for the Babbacombe Nursing Hospital for Sick and Incurable Children. On February 15th identically the same advertisement appeared in the *Nursing Record*. Our advertisement contained a printer's error, the figures "42" being printed instead of "12," through an indistinctness in the manuscript. This error is faithfully reproduced in the *Nursing Record* of 15th inst., and the advertiser, on being referred to, has written in reply that there was no advertisement sent to the *Nursing Record*.

The second case is that of an advertisement for under nurses at Berrywood Asylum, Northampton, which first appeared in The Hospital on 15th inst., and was exactly recopied into the *Nursing Record* on February 22nd. The medical superintendent, in reply to our inquiry, writes: "I know nothing of the advertisement in the *Nursing Record*."

Our objection to this system of piracy is that it causes trouble to our advertisers and grievous injury to innocent nurses. The advertiser's annoyance is due to the receipt of a number of applications *after* the vacancy has been filled up, and the injury to the nurse is caused by the loss of time expended on answering an advertisement under such circumstances, and the consequent disappointment. Nor is this all; for a nurse who answers an obsolete (pirated) advertisement appearing in the *Nursing Record* may frequently be deterred thereby from exerting herself to gain employment in other directions in the belief that the eligible and advantageous appointment, the advertisement of which has only just come before her, is still vacant.

We are confident that the opinion of the whole nursing world will condemn this system of pirated advertisements.

### Presentation.

Melville Street Nurses' Institution, Edinburgh.—Nurse M. Johnston, on leaving this institution to be married, was presented with a silver tea service by her fellow nurses, and also received a handsome eiderdown quilt from the institution, the presentation being made by Mr. Young, the treasurer.

### Royal National Pension Fund for Nurses.

### THE NURSES' GIFT TO H.R.H. THE PRINCESS MAUD OF WALES.

When the fact was announced that H.R.H. the Princess Maud of Wales was engaged to marry her Royal cousin, Prince Charles of Denmark, we received numerous letters from nurses of the Royal National Pension Fund expressing an earnest wish to be allowed to offer a wedding gift to the daughter of their beloved President. We readily consented to open a contribution list in our columns, suggesting that subscriptions should consist of one shilling only, feeling that thus the less well-to-do might join in the pleasant project with those of ampler means without sacrifice to themselves. Two ladies of the committee of the Junius S. Morgan Benevolent Fund kindly allowed the money, as it was received, to be entered in their names, and no appeal of any sort was issued, so that the collection was made free of all expense, and is entirely spontaneous on the part of the nurses. We feel sure the Royal President of the Fund would feel gratified could she read the letters full of loyalty, gratitude, and affection which accompanied the offerings of the nurses. One member writes, "I am glad to think we are allowed to show a little of our gratitude to 'our Princess' in giving a present to H.R.H. Princess Maud," and most of the letters are full of the same sentiments.

We asked the nurses to intimate what form they desired the gift to take. The answers were very varied, and so we adopted the apt suggestion of one who wrote, "I think it would be best to take our kind and gracious President into the secret." The result was that a beautiful tea-table, selected by the ladies of the Benevolent Fund Committee, is now ready for the Princess's acceptance. But before Her Royal Highness receives it, we feel sure that the nurses who are presenting the gift would like to see it themselves. Mr. Albert Barker, the maker, of 5, Bond Street, has signified his willingness to allow nurse donors and their friends to inspect the table at his premises on any day between February 28th and March 13th. We give an illustration of the table that those who are too far distant to visit Bond Street may gain some idea of the appearance of their gift. The idea must of necessity be very inadequate, for the beautiful colouring of the inlaid table, the charming delicacy of the hand-painted china, and the elegance of the silver accessories cannot be reproduced. Each of the eight teacups, which are garlanded with tiny roses, possesses a beautiful little spoon, the handle of which is twisted into an appropriate true lover's knot. The tea set stands in a strongly-framed glass tray.

The Nurses' Present to the Princess Maud.

But the table itself is no ordinary table. It is what Mr. Barker, the clever designer, calls a "surprise table." That is to say, the flaps which are shown spread out now that the table is open, close towards the middle, and as they close the tea-tray gradually sinks down into the body of the table, and the closed flaps show no evidence of any tea equipage existing. In opening, the tray rises gradually as it disappeared. The flap, on which a plate is shown resting in the illustration, slides out of sight also. The construction is convenient and attractive. It raises the present above the level of ordinary tea-tables, and we trust that nurses of the Pension Fund will be satisfied with the choice that has been made for them, and that it will fulfil the desire which a nurse expresses in her letter that it will be "in daily use" in the new home of the Princess.

### Opening of Wards at St. Thomas's Hospital.

### A LETTER FROM MISS NIGHTINGALE.

The following letter was received from Miss Nightingale by Mr. Wainwright, treasurer of St. Thomas's Hospital, and read by him at the opening of the "Beatrice" and "Florence" wards last week: "My dear Sir,—It gives us all joy, and patients especially, your reopening of two much-wanted wards, lying empty for want of funds, through no fault of their own. These wards also contain some of the latest improvements. And I hope that all the money still wanted, which you desire and deserve, will come in. I beg to send my £100, wishing it were much larger, for the benefit is great, not only to the bodies of the patients, but towards rendering those bodies able to tread a nobler, more useful course in life by the practical lessons they learn in the wards of order, kindness, and moderation, or self-discipline. This is especially the case with the children. It is delightful when a hospital is a school of good morals to the patients as well as a training school for nurses and for students. And such is St. Thomas's Hospital. We rejoice that our president, the Duke of Connaught, is to perform the ceremony of opening the wards.—I beg to remain, my dear Sir, yours very faithfully, Florence Nightingale."

### On Certain Aspects of the Nursing Question as Seen in England and Germany.

By a Certificated Midwife.

### II.—ENGLAND AND HER WAYS.

The sort of women who go in for monthly nursing in England are generally but poorly educated, chiefly the widows of small tradesmen, or domestic servants who aspire to a more independent life, and who think, rightly or wrongly, that they have a talent for "smoothing pillows" and "cheerful conversation." There are a few ladies who want to make a livelihood, and a few more who take up the profession from charitable motives, and intend to work amongst the poor. But as a rule those who are to attend on ladies know absolutely nothing, when they enter on their training, of the framework of the human body, its organs, or its functions; still less how they may be altered or interfered with in the process of childbirth. Scarcely one of them has even seen a birth, though they may have borne children themselves. What they have to learn, then during their training—what they certainly ought to learn—and what they are credited with knowing by the fact of their certificate is—First, how to meet the wants of a mother in a normal, and how to detect the signs of an abnormal case; second, how to care properly for a healthy infant, and how to know and deal with an unhealthy or delicate one; third, how to attend on the medical man when he is there, what conditions make it imperative that he should be summoned, and how best to act when he is unfortunately absent. Upon this knowledge there should be superimposed nice ways, handiness, and refinement of speech and manner. The cost of training varies, but it can hardly be thought that £6 or even £8 is too much to pay for such knowledge and experience. But four, six, or even eight weeks is far too short a time to acquire it in. What, moreover, is the routine of this training and instruction in the principal lying-in hospitals? A would-be nurse, knowing nothing save the fee she is to pay, and the costume she is to wear, arrives at the hospital on the day appointed. She is shown her bed, told the hours for the doctor's visits, and of meals, and waits her turn to "see cases." Meanwhile, she buys a "Manual of Midwifery and Monthly Nursing," as advised, and sits down to read it, very likely not understanding a single sentence. All the Latin words used in the book are meaningless to her. Pelvis, uterus, gestation, are just gibberish, and nothing more, and in England it seems that the facts and processes of childbirth cannot be written without being wrapped up in these technical terms. The next day she attends, perhaps, a lecture, which the visiting physician gives two or three times a week, if possible. He begins a course say on the first of the month. Out of, perhaps, twenty nurses present five have newly arrived, five are leaving in a few days, when five fresh ones will step into their places, and be followed by five more the week after. The able lecturer has prepared a sequence of instruction, and does his best to present it in its simplest form. But how about his hearers? Those who have never heard the beginning are sorely puzzled by the final one of the course; those who begin in the middle do not grasp a single idea correctly. The lecturer himself, seldom seeing the same faces on two following weeks, rarely learns to distinguish one pupil from another, and thus, unable to test their intelligence by personal questions, or to fathom their difficulties, often in despair reduces his instruction (which would be of inestimable value could they take it in) from definite information to indefinite platitudes. And the practical training follows suit. Not knowing anything about the paraphernalia of a "Labour Ward," the novice is called in due course to witness a birth. She gets nervous and hot with the cries and groans of the patient, thinks the midwife heartless, says "poor dear!" out loud, and gets snubbed by the "resident." She blushes and feels she is doing something indecent when she gets near the bed and tries to see what is taking place, and gives way modestly to the young surgeon or, perhaps, a student or two who are also getting their experience. Later on she is given charge of one, two, or three new-born babes and learns how to wash and dress them in a certain fashion and how to attend to the mothers according to hospital routine. She hears a good deal of vulgar talk, and frequently acquires a habit of gossip about things best buried in silence. When she leaves at the end of her appointed weeks she has learned to answer by rote certain pass questions, knows certain things a medical man will require from her in attending on a private case, and has acquired a very uncertain amount of knowledge as to the use of disinfectants and stimulants. On her certificate is entered the number of cases she has seen, sometimes even the number of births that have place taken in the hospital whether witnessed or not; and with a very clear idea in her head that she must always wear nice white dresses if she wishes to nurse real ladies, and a very hazy one with regard to pyæmia and ophthalmia the hospital door shuts behind her and she is launched on her career. She may or may not be a very sensible woman who will do well in it. She may or may not be a very inefficient nurse who will bring discredit on her calling, that is as Providence has ordained. The authorities are not to blame. They have done all they could under the circumstances. The nurses' fees help the charity, and though they would learn more if there were not so many taken in, is it in human nature to send away superfluous ones? They would learn more, too, if there was not such a hurry and skurry over most or many of the patients, so that the chief object is to get the mother safely into bed before the child is born, and not to teach the pupils. But the constitution of the hospital forbids entrance to a mother till she is in labour, and how can the hurry be helped? She would learn more, too, if the term's complement of pupils were admitted all at once. But Englishwomen are free, and free will is a most uncertain factor to work with. It is a haphazard system of training, if system or training it can be named; but it has its roots deep in the character and idiosyncrasy of the Briton. And on the whole English mothers are content with its results. As for me, after the completion of my training, I remained on in the same hospital as "resident," and taught to the best of my ability a long succession of pupils. But I felt a constantly growing dissatisfaction with the inefficiency of the organisation under which I worked, as well as deep conviction that the death-rate was higher than it ought to be. Accordingly I gave up my post, packed up my box, and started for the Continent, intending to go to Vienna. But the Fates ordered it otherwise, and it was eventually at Stuttgart that I gained my first experience of those foreign ways which I set out to learn. Later on I entered myself as a pupil both at Vienna and Dresden, the midwifery schools of these cities bearing a high reputation. What I learned in Germany will be set forth in my next article.

### Novelties for Nurses.

### SWEETMEATS.

We were pleased once before to draw the attention of our readers to the very excellent cream caramels of Messrs. Clarke, Nicholls, and Co. The specimens sent to us recently enable us to state that the caramels maintain their character for excellence, and we can once again recommend them with confidence as a delicious and wholesome sweetmeat.

### Some Experiences of a Lecturer on Sanitation.

I WAS looking out for some work to do. My friends urged me to try sanitation, saying that that was one of the few occupations in which the supply did not exceed the demand. The County Council and one or two societies were said to be in want of lecturers on sanitation and hygiene, and there was a strong feeling that more women inspectors of factories and workshops were needed and would shortly be appointed. This seemed to be the kind of employment for which I was looking; I accordingly joined a class then being formed, and entered my name also for a series of lectures on sanitation and hygiene.

I worked hard at filtering beds, drains, ventilation, disposal of sewage, chimneys, subsoils, traps and water-seals, dustbins and overflow pipes, making many drawings of what to avoid and what to choose. I became a scourge to my friends in the matter of room ventilation, and discovered that most of the ordinary domestic filters were no better than germ traps. The result of shaking one of them (which by the directions on the filter itself one is specially requested not to do) proclaimed the fact that creatures resembling crocodiles and sea-serpents were found in great abundance reposing in the bed of the filter.

After learning many things about food and milk, I began to read up the Acts of Parliament relating to the matter, for I knew that if I wished for an inspectorship I must have all those relating to sanitary matters and "nuisances" at my finger ends. So I learnt how to drain and arrange slaughter-houses, how to detect bad meat, in spite of the butcher having removed the incriminating parts, and what were the names and favourite dwelling places of the animalculæ that seemed to infest most animals.

In due time I presented myself for examination at South Kensington, when I found to my consternation that no student might answer the paper on hygiene unless he had previously satisfied the examiners in physiology. So I had to depart, having paid my fees uselessly.

No one seemed able to explain why it was necessary to know all about the structure and functions of the body before a pupil was competent to lecture on "ground air and drain pipes," but so it was, and I next tried to master the contents of a bulky volume on physiology.

The long Latin names were as nightmares, and after I had mastered them the difficulty was to remember to what parts they applied. I had no natural liking for a minute inspection of the inside of the human body, so I was glad to hear that at the Parkes Museum a certificate could be obtained without taking up either physiology or anatomy.

After obtaining my certificate I soon got an appointment as lecturer on sanitation and hygiene in the North of England, and I started on my tour through a rural part of the county.

It appeared not to be considered seemly that I should stay at the village inn, so I was generally quartered on some superior cottager, where my fate usually seemed to be to occupy the room "in which my daughter died!" Oh! the discomfort of these amateur lodgings! Roughing it is all very well when out for a holiday, but I was at work, and I found that my lectures and "conversations" required all my powers, and these powers had very little chance of being kept at a respectable level with the poor food and miserable accommodation with which I had to content myself when on most of my tours.

A description of one place may equally serve as a description of all. On arriving at my village I was made "kindly welcome" by the good wife, and given the seat of honour on the settle (the parlour was so full of wool mats and paper ornaments that I begged to be allowed to sit in the kitchen). My bedroom looked clean, but oh, the bed! Lumps of some hard stuff were under my shoulders, and other lumps were under me at intervals everywhere else. Through the long hours I used to wonder which were the worst, the lumps or the intervals!

A curious smell, a mixture of apples and dry rot, pervaded everything, and the window only remained open a tiny space when propped up with my boots. In the morning the refreshment of a cold tub was an impossibility. I found a small basin placed for me on a chair, whereupon I begged that I might come down and wash at the sink in a tin bucket. As my hostesses were generally widows this arrangement was feasible.

My food was always eggs or bacon, or both together; once or twice I tried "butcher meat," but the experiment was not a success.

I found that the lectures themselves were considered by most of those who attended them as dealing with "useless fads." Most of the modern appliances were regarded with disfavour, as calculated to get out of order, and as very complicated when compared with their present primitive contrivances, which, they assured me, satisfied all their needs. "I know it's the fashion to say we live unwholesomely," said a quite intelligent-looking young farmer to me; "but we've thriven fine on the old system. Look at me, m'am!" And certainly he was as fine and healthy a looking piece of humanity as one would wish to see.

The water supply was a continual source of discussion. The inhabitants of the rural districts usually had to fetch their water either from the village pump or from a neighbouring brook, that was conveniently made to flow into a stone trough into which they could dip their cans and buckets.

As these troughs were by the roadside they became foul very quickly, and it was small wonder that the natives preferred drinking either beer or tea to this uninviting cold water.

In fact I was often tempted to give a lecture on "The Dangers of (so called) Temperance!"

I had many battles over pumps and pigstyes.

By a curious fatality the pigstyes seemed to be always placed at the top of the garden, from which point of vantage their contents could gently and unceasingly filter through into the well, which was, as invariably, placed a few paces below.

A few of my people thought "there might be some truth in what I said about the stuff soaking in, for it stood to reason," and so to "oblige me" they moved their styes—an effort at advancement and improvement for which I, of course, had to be personally grateful!

My "social" experiences were often entertaining. The local celebrities usually invited me to afternoon tea. Many of them were not quite sure how to treat me, thinking that I "could not be very nice, really, to take up such unpleasant subjects," and so they usually confined their conversation to such safe topics as the weather.

The continual moving from place to place made it a somewhat trying life, but I abandoned it with regret.

### Minor Appointments.

WILTS COUNTY ASYLUM, DEVIZES.—Miss Jessie Bissett has been appointed Head Nurse of this asylum. Miss Bissett was trained at Garlands and Merryflats, holds the certificate of the Medico-Psychological Association, and is leaving the Smithston Asylum, Greenock, where she has been chief nurse to take up her new appointment at Devizes.

### Talks About Food.

### DRINK.

We are so accustomed to making a distinction between food and drink, and talking as though one were the direct opposite of the other, that to some of our readers it may very likely seem almost absurd to write an article on food, and then to entitle it "Drink." A moment's consideration, however, will show that this distinction, however convenient in common parlance, has, from a physiological point of view, no meaning at all; indeed, the fact that we constantly use the expressions, "solid food," and "liquid food," will show us the truth of this at once. Many of our drinks, as we know, are rich in solid matter in a state of suspension; others, such as tea, may well be called nerve food; and even water may be called a food, for, physiologically speaking, all things are food which contribute to the nutrition and growth of the body.

For practical purposes, however, this definition will not help us much; and we will, therefore, simply say that drinking serves one or more of three distinct purposes: that is to say, it may supply—firstly, mere liquid (which we do not generally consider food at all); secondly, nerve food; and thirdly, solid matter for the building up of the body. It is well to bear this in mind; for, when the suitability of various drinks is discussed, the first question asked should be this: What purpose is this drink meant to serve? If a sufficiency of solid food has been taken, it is wasteful, and often injurious, as Sir Henry Thompson has pointed out, to take such a drink, for example, as milk; and if, on the other hand, some laborious task has to be accomplished, and solid food is not easily obtainable, it would be foolish to stick to the lemonade or water which would be most suitable in the former case.

Both rich and poor are far too much afraid of water, the natural beverage of man. We verily believe that there are large numbers of persons among the upper classes who do not know the taste—or rather, the no-taste—of it, having always been taught that it must have something "put to it" before it is fit to drink. This is a great pity, for a draught of bright, clear water is a real luxury to those who have not been taught to shun it; and when the sensation of thirst—that is, of the want of liquid matter in the system—is felt, why not gratify it in the natural way, without an admixture of some unwanted element?

And it is much the same, as has been said, with the working classes. The moment thirst is felt, they fly to beer as a matter of course, and consume, if they are engaged in hard work, almost incredible quantities of it. That they make a serious mistake in so doing may be seen by the "boozy" condition of many a harvester, and by the drunken brawls which not unfrequently take place in the harvest fields. During last harvest one of these cases came up before the magistrates, and after the condition of one witness after another to the alleged assault had been spoken to, the magistrate at last asked indignantly, "Why did you not bring as a witness the one sober man in the field?" In such crucial cases as the employment of men in a highly heated atmosphere—near a furnace or boiler, for instance—even the men themselves have to confess that alcoholic drinks are hurtful, and that no drink is so good as water with a little oatmeal thrown into it to take off the rawness; and an able physician has declared his belief that the emphysema and black deposit so often found in the lungs of certain of these artisans is in no small measure due to their persistent use of alcohol and neglect of its antagonist, water.

This being so, employers of labour should do all in their power to make it easy for their men to obtain drinks other than alcoholic. For men working in extreme heat, nothing can be better, as has been said, than water with a dash of oatmeal; but where other work is concerned, there is no reason why drinks containing more nourishment should not be used; indeed, there is great reason why they should. A labourer—a farm labourer, at any rate—is seldom over-fed; and a drink which should serve the purpose of a food as well will be better for him, and will have a far better chance of proving acceptable, than one which merely supplies him with liquid. Tea or coffee, with plenty of milk and sugar, and drunk either hot or cold, would be a far more suitable drink than beer; and better still, if nourishment is needed, are the queerly named stokos and cokos, directions for making which are given in one of the Church of England Temperance Society's leaflets, the latter being made of oatmeal, cocoa, and sugar, upon which boiling water is poured, and the former of oatmeal and sugar only, with a little flavouring of lemon. These may be drunk hot or cold, and we have found them to be highly appreciated in the hay and harvest field; indeed, so zealous was a certain cokos-making lady of our acquaintance, and so responsive were the men, that towards the end of the first day of it one or two of them announced that "they couldn't work no more—they fared to feel sa full!" Of which the moral would seem to be that some other drink, of a thinner and less "filling" character, should be tried alongside of the cokos.

We feel sure that farmers would do much better by their men, and by themselves too, if they took some trouble to promote temperance in the harvest-field, instead of practically giving, as some of them do, a part of the men's wages in malt and hops—surely a moral evasion of the Truck Act. Milk, again, would be a capital drink for a hard-working and thirsty man, and one that some farmers could very easily supply; and buttermilk is by no means to be despised, being lighter than milk, and nourishing withal; indeed, a writer on food has declared that to see it given to the pigs "makes the philanthropist's heart bleed." "Gingerette," again, is very useful in a different way, taking the place of the hot brandy and water to which recourse is had after a wet walk or a chill. We have not forgotten the look of radiant satisfaction with which a labourer rubbed himself down after a draught of hot gingerette and water, remarking as he did so, "That glow on the stomach, I do believe."

We need hardly say that cocoa is an excellent drink, pre-eminently for the poor, whose means of obtaining nourishment are limited. It contains the same properties as tea and coffee, though in a milder degree, and is also rich in nitrogenous matter and cacao butter. It might with great advantage displace, at one of the meals, at any rate, the ever present tea of the working classes, which, with its frequent lack of milk, and in its stewed condition, is good for very little. It is really melancholy to see how tea is ruined by cottage housewives, being stewed for hours by the fire, until it becomes a vile decoction of tannin. A doctor of our acquaintance declares that when he is unexpectedly detained for hours by some cottage patient, and reduced to accepting such hospitality as can be offered, he is invariably made ill by the stewed decoction of tea-leaves presented to him. "But then," he adds, with a comical shudder, "as a matter of fact, I believe it's not tea, but sloe leaves!"

Teetotallers, like vegetarians, have done very useful work in combating popular delusions and errors. But there yet remains an enormous amount of work to be done; and if we cannot see our way to denouncing beer as poison, and the use of alcohol as a sin, we may yet work alongside of the teetotallers in recommending to notice articles which have hitherto been more or less despised. Beer and spirits are too much pushed into notice already—even a strong anti-teetotaller must admit that; and if men and women whom the alcohol-loving world calls "sensible," will only help to give other drinks their fair chance, we quite believe that they will meet with success, where the rabid teetotaller can make no way whatever.

### Everybody's Opinion.

[Correspondence on all subjects is invited, but we cannot in any way be responsible for the opinions expressed by our correspondents. No communications can be entertained if the name and address of the correspondent is not given, or unless one side of the paper only be written on.]

### PRIVATE NURSING.

NURSE BERTHA WOOD writes: I have read with interest the good advice given by "Sister Grace" to those who really wish to become hospital nurses. It is not the easy life that many young women think. I have often heard a nurse say that a general servant had a much better time than herself, so that I was sorry to see "Nurse Kirkman's" reply, for there are many upper servants educated and refined enough to be able to read and even to do the correspondence; but the nurse is wanted for her hospital knowledge and training, not as a reader or secretary. I knew of a young lady who left the hospital because she had to attend the baby of a patient; the child was still being nursed by the mother. How can such young women expect to be good nurses if they do not want to learn the practical part of nursing as well as the theory? I once had a case where the mother was operated upon, and I not only had to attend her, but I had to cook the dinner for the father and children. Of course they were poor people, but if I had not done it I should have felt myself a poor sort of a woman, much less a nurse. If "Nurse Kirkman" went to Australia, or any of the young British Colonies, or come out here to Brazil, she would have to do some very rough work. The matrons and sisters should all be gentlewomen, and as there are so many ladies of rank and even title joining hospitals now, there should be no difficulty in supplying these posts. But I feel sure there are a number of respectable young women who, as "Sister Grace" puts it, are born nurses, but who, because they happen not to have received a first-class education, are considered not capable. To my mind conscientiousness and love of her work comes before education with a sick nurse.

### HELPERS OR RIVALS.

NURSE ANNE E. DEMEL writes: This rather new and unexpected side to the trained nurse question which was mentioned in last week's HOSPITAL, as to whether nurses are to be considered loyal helpers or rivals to the doctor, does indeed call for serious and judicious inquiry. A really well-trained nurse knows it is her first duty conscientiously to carry out the instructions of the medical man, of whose case she has charge, with unquestioning obedience and to the letter, impressing those around her with the importance of so doing. The doctor prescribes orders, and the nurse receiving his instructions herself, carries them out as only a well-trained nurse can do; thought of self will not intrude where duties so grave are concerned. When following on these lines "the little learning" she is supposed to possess will not "upset her mental balance," but will materially help her in the faithful discharge of her duties as nurse. Then what an immense amount of worry a really good nurse may save the busy practitioner by attending to what untrained persons would call small or unimportant details in connection with the patient; also by giving exact reports as to any change in patient's condition, being very careful in her own personal behaviour towards the patient's relations and friends, courteous and kind to all, giving as little trouble as possible to the often harassed servants of the household. Again, as for "scaring" a patient with visions of possible peritonitis where spasms is the ailment, &c., these ideas can only be the thoughtless suggestions of a very ignorant person; a well-trained nurse would soothe her patient, and help to assuage the pain with such remedies the doctor had himself ordered. Then the idea that nurses fresh from hospital have a "contempt" for the general practitioner is surely a very great mistake. Also the male nurse who "carries his knowledge easily, and by means of which becomes more clearly aware of his own ignorance," let the doctors employ him oftener to perform those duties for men patients which a woman should never be called upon to do. It is to be feared, however, that the male nurse will tread closer on the doctor's heels—be more truly a rival in the sick-room than the much-found-fault-with trained woman nurse.

### Practical Points.

*A Nurse writes: Can you give me any information about the Alexandra Hospital for Children with Hip Disease, Queen Square? I have been asked about it by a patient who is inclined to subscribe, as a thank-offering for recovery from an accident, in response to the plea for help which has lately appeared in the papers, and who wishes to be sure that the institution is a deserving and worthy one.*

*Another correspondent writes: Can you tell me anything of the Hospital for Children with Hip Disease in Queen Square? I see a piteous appeal for help, and would like to know if it is well managed and a deserving object of charity.*

The Alexandra Hospital is an excellent institution, and help may well be given to it with both hands by the grateful patient or any other charitably disposed person. A personal visit will convince everybody of the excellence of the management under Miss Moore, who is untiring in her efforts for the comfort and relief of those sick children for whom there is no place elsewhere. Such a visit, too, is the best way to find out how great is the need for this hospital, and how urgently the aid asked for is required.

### Where to Go.

ST. MARTIN'S TOWN HALL.—The Ladies' Guitar and Mandoline Band (conductor, Signor Francia) will give an afternoon concert, in aid of the Waifs and Strays Society, on Wednesday, March 18th, at St. Martin's Town Hall, Trafalgar Square. The concert will be under the immediate patronage of Her Royal Highness the Duchess of York.

GREAT NORTHERN CENTRAL HOSPITAL.—A grand morning concert is announced to take place at the Queen's Hall, Langham Place, on Thursday, June 11th, in aid of the Ladies' Association Fund of the Great Northern Central Hospital. The concert is under the special patronage of T.R.H. the Duke and Duchess of York, the Duchess of Teck, and the Duchess of Albany. Tickets at 21s., 10s. 6d., 7s. 6d., 5s., and 2s. 6d. Any information supplied by Mrs. Harkness, hon. secretary of the Ladies' Association, at the Great Northern Central Hospital, Holloway Road, N., or by C. C. Osborne, Esq., 1, Stratton Street, W.

### Wants and Workers.

[THE attention of correspondents is directed to the fact that "Helps in Sickness and to Health" (Scientific Press, 428, Strand) will enable them promptly to find the most suitable accommodation for difficult or special cases.]

NURSING ABROAD.—Replying to a query in "Wants and Workers" last week under this head, a correspondent writes: "With regard to private nursing at Gibraltar, there would not be any opening at present, unless a nurse had private means and could afford to wait. There is a sister attached to the Colonial Hospital available for private cases, and there is plenty of work for one, but hardly for a second nurse. A knowledge of midwifery would be an essential qualification."

LAST week attention was drawn under this heading to the opportunity for rest and change offered to nurses by the owner of a pleasant little house in Cheshire, application to be made to Nurse Mary, 11, Clarence Road, Teddington, Middlesex. We regret that there was an error in terms mentioned. These should have been given as 8s. 6d. weekly, or 8s. each to two nurses willing to share one room.

### The Book World for Women and Nurses.

[We invite Correspondence, Criticism, Enquiries, and Notes on Books likely to interest Women and Nurses. Address, Editor, The Hospital (Nurses' Book World), 428, Strand, W.C.]

### TWO NEW PERIODICAL PUBLICATIONS.

Two new and delightfully got up works, to be published in weekly parts, have just been brought before our notice. One of these, "Cassell's History of England," is to be issued in 52 weekly parts, each number containing 96 pages of well-printed matter, profusely illustrated. This People's Edition ought to be a real boon to those who are students of English history, and there are many, too, among us who wish to refresh our memories on these points. Nos. 1 and 2 of this delightful work are now ready, and can be supplied at the modest sum of 6d. each through any bookseller in the United Kingdom. The other publication to which we referred as now being issued in weekly form, "The Portfolio of Palestine Pictures," we are informed, is a reissue, by special request, of "The Earthly Footsteps of the Man of Galilee," of which over a million copies were sold last year. We cannot be surprised at the success of this volume, for it is based on entirely original lines, and is got up in an exceptionally fine manner. Bishop Vincent, LL.D., and the Rev. W. Lee, D.D., contribute the letterpress, whilst the special artist is Mr. Robert E. M. Bain. This wonderful sixpennyworth is to be obtained from the offices of the *Christian Globe*, 185, Fleet Street, E.C.

### Notes and Queries.

**Queries.**

(160) *Training.*—Will you kindly give me your advice? I am seventeen, and wish, when old enough, to enter a children's hospital for training. Could I enter any institution for children, perhaps a convalescent home, now, until that time, and so become acquainted with the work?—*Would-be Pro.*

(161) *Gibraltar.*—Can you tell me if previous training in an English hospital is necessary in order to be eligible for a vacancy on the nursing staff of the Civil Hospital at Gibraltar?—*K. N.*

(162) *Correspondence Classes.*—Can you give me Miss Lamport's address?—*Nurse A.*

(163) *Service Abroad.*—Will you kindly tell me of any paper where ladies advertise for servants to go abroad with them?—*H. L.*

(164) *Asylum Training.*—Will you tell me at what asylums attendants are trained for the certificate of the Medico-Psychological Association?—*A. D.*

(165) *Books for Nurses.*—Can you let me know the publishers and prices of these books?: Quain's "Dictionary of Medicine," Percy Lewis's "Theory and Practice of Nursing," and Taylor's "Practice of Medicine."—*Queen's Nurse.*

(166) *Hypnotism.*—Can you tell me what is the best book upon hypnotism, and also give me the name of a good work upon melancholia and its treatment?—*Ingaborge.*

**Answers.**

"An Old Reader," "Alice," and "Mistress" are reminded that queries without address or signature cannot be answered in this column.

(160) *Training (Would-be Pro.).*—Eighteen is quite the earliest age at which you would be likely to obtain admission into any institution such as you mention, and that is quite time enough. At most children's hospitals the age limit for probationers is twenty to twenty-three, sometimes, though rarely, nineteen. Get "How to Become a Nurse" (Scientific Press, 428, Strand, W.C.), in which you will find every information and complete lists of hospitals, with particulars of training, terms, &c., for future guidance, and make up your mind to wait patiently till you have gained a little more age and experience.

(161) *Gibraltar (K. N.).*—Only thoroughly-trained, certificated nurses are engaged for the Civil or Colonial Hospital at Gibraltar, and these are generally from the London Hospital, Whitechapel, engaged through the Crown Agent's office. There is not likely to be any vacancy for a year or two.

(162) *Correspondence Classes (Nurse A.)*—Miss Lamport's address is 52, St. John's Wood Road. It was given in this column on January 25th.

(163) *Service Abroad (H.L.).*—There are frequently advertisements in the *Morning Post* which might answer your purpose.

(164) *Asylum Training (A. D.).*—Write to the secretary of the Medico-Psychological Association, 11, Chandos Street, London, W.

(165) *Books for Nurses (Queen's Nurse).*—You can obtain any of the books you name through the Scientific Press, 428, Strand. Quain's "Dictionary," published price 31s. 6d., Longmans; Lewis's "Theory and Practice of Nursing," 3s. 6d., Scientific Press; Taylor's "Practice of Medicine," 15s., J. and A. Churchill.

(166) *Hypnotism (Ingaborge).*—(1) "Hypnotism," by Albert Moll (of Berlin), London, Walter Scott, 1891; (2) there is an article on melancholia in Quain's "Dictionary of Medicine," by Dr. Blandford, and in Hack Tuke's "Dictionary of Psychological Medicine" (London, J. and A. Churchill, 1892), there is one by Charles Mercer. There is a fairly full account of melancholia in "Insanity and its Treatment," by G. Fielding Blandford, M.D., Edinburgh, Oliver and Boyd, 1884.

### Reading to the Sick.

### LIFE'S PURPOSE.

**Motto.**

Every man's task is his life-preserver. The conviction that his work is dear to God and cannot be spared defends him.—*Emerson.*

**Verses.**

Be sure, no earnest work
Of any honest creature—howbeit weak,
Imperfect, ill-adapted—fails so much,
It is not gathered as a grain of sand
To enlarge the sum of human action used
For carrying out God's end! —*E. B. Browning.*

Death closes all; but something ere the end,
Some work of noble note may yet be done!
—*Clough.*

Father, I know that all my life
Is portioned out for me,
And the changes that will surely come
I do not fear to see;
But I ask Thee for a present mind,
Intent on serving Thee.

Wherever in the world I am,
In whatsoe'er estate,
I have a fellowship with hearts
To keep and cultivate;
And a work of lowly love to do
For the Lord on whom I wait.

**Reading.**

"The presence of the Lord is the secret of all strength and comfort. A man cannot know what a serious thing his life is until he sees that he is ever with Christ in it; that for a little space he is called out of the throng, to do solemnly his own part before the face of the Son of God. As he grows to perceive this, all becomes earnest around him. Life is a real thing to such an one; real in its deep heart of joy, real in its connections, its actions, and its changes. And with this sense of reality is closely joined a blessed freedom and gladness, such as that which blesses our innocent childhood. The strength of a constant will, the artlessness of an open heart, the sense of safety, the gladness of a filial confidence, and the sparkling play of the unclouded affections—these are the blessed fruits of leading a life in the remembrance of the constant presence of our Lord."—*Wilberforce.*

Each one of us was "sent from God" into this world. If we are sent from God it is on some definite errand. God has a plan, a purpose for each life. No immortal soul ever came by accident into this world, and none ever came without a mission. We ought to think of this. People sometimes suppose that such men as Moses and John and Paul were exceptions. They had their own specific mission; God sent them on very definite errands. But surely we common people are not sent from God in the same sense. We never saw God in a burning bush, nor received our commission directly from His lips. No angel came before our birth to announce what we were to be and to do in this world. We had no revelation of bright glory smiting us down in blindness, yet we are "sent from God," every one of us, and have as definite a work allotted to us as had Moses or John or Paul. Are we living out God's thought for us, what He had in view when He made us and sent us hither? Are we doing in this world what He wants us to do? These are important questions, and we should not stop short of honest answers to them, for we shall have to account to God at the end for the way we have fulfilled our mission. Any life is a failure which does not accomplish that which God sent it into the world to do. We find our work and our mission by simple obedience to God and submission to Him.—*H. J. Miller, D.D.*

## Figures and Tables

**Figure f1:**